# Assessment of the influence of 660 and 808-nm PBM treatments on mitochondrial oxygen consumption of MG-63 osteoblast: a 3D cell culture study

**DOI:** 10.1007/s10103-025-04349-3

**Published:** 2025-02-12

**Authors:** Simone Sleep, Deanne Hryciw, Jennifer Gunter, Praveen Arany, Nifty Tomy, Roy George

**Affiliations:** 1 Griffith University, Southport, Australia; 2 Queensland University of Technology, Translational Research Institute, Brisbane, Australia; 3 University at Buffalo, Buffalo, USA; 4 National Centre for Disease Informatics and Research, Bangalore, India

**Keywords:** Osteoblasts, Lasers, Bio photonics, Cell signalling, Molecular biology, Photo initiators

## Abstract

This study explores the dose-dependent effects of 660-nm and 808-nm photobiomodulation (PBM) on mitochondrial oxygen respiration rate activity in MG-63 osteoblast cells using an innovative 3D in vitro spheroid model. MG-63 osteoblast cells were grown to 80% confluence and seeded in fish gelatin hydrogel (LunaGel™) to form 3D spheroids within 3–7 days. Spheroids were seeded on Seahorse microplates and incubated in a LunacrossLinker™ (visible light crosslinking system) for 2 min to give hydrogel a mid-stiffness of 3.5 kPa. Cells were exposed to PBM either 660-nm or 808-nm at panel setting of 5 J/cm^2^ and 15 J/cm^2^ and then assessed immediate (15 min before analysing) and 24 h time points. Mitochondrial activity was determined using an XFe96 Seahorse analyzer. Data distribution was assessed, and parametric or non-parametric tests and compared the mitochondrial respiratory capacity across different experimental conditions. The study indicated that 660-nm and 808-nm PBM could modulate mitochondrial functions in osteoblasts. The maximal respiratory rate for the fluency assessed at 808-nm wavelength was increased when cells were assessed immediate post. Interestingly, the 660-nm PBM-treated cells showed a decrease in oxygen consumption rate (OCR) at the basal and maximal bioenergetic state at all time points (immediate and 24 h.) and fluency compared to the untreated control. The effects of 660-nm and 808-nm wavelengths on osteoblast mitochondrial function suggest that PBM demonstrates differential modulation of osteoblast metabolism and bioenergetics depending on the wavelength. These findings have practical implications in both research and clinical settings, offering insights into selecting specific wavelengths for therapeutic applications.

**Supplementary Information:**

The online version contains supplementary material available at 10.1007/s10103-025-04349-3.

## Introduction

Photobiomodulation (PBM) Therapy, also known as Low-Level Laser Therapy (LLLT), is the application of red and near-infrared light over tissues to enhance healing, mitochondrial activity, reduce inflammation, and manage pain [1]. The near-infrared (808 to 1000-nm) light source can be produced by laser or (Light Emitting Diode) LEDs. It is reported that PBM dose and wavelength could influence cell and mitochondrial metabolism [2]. Understanding mitochondrial metabolism is crucial for our study because PBM directly affects the photo-acceptors found in the mitochondria, potentially impacting their function and overall cellular health.

PBM is reported to influence or modulate cellular behaviour, hence enhancing tissue repair mechanisms [3]. Khan and Arany (2015) stated that PBM impacts mitochondrial respiration [4]. Specifically, Bikmulina et al. (2020b) noted that PBM at visible (400 to 700-nm) and near-infrared (700 to 1100-nm) wavelengths increased mitochondrial respiration and counteract the inhibitory effects of rotenone [5]. Rotenone is known to induce cell death via inhibiting the mitochondrial respiratory chain complex I [6]. PBM appears to promote cell survival and augment mitochondrial oxygen uptake, offering potential benefits for various disorders [7]. Wang et al. (2017) reported that 808-nm primarily stimulated the mitochondrial cytochrome c oxidase (CCO), resulting in cell activation [16]. Evidence also suggests that the action of PBM on target tissue can be immediate with multiple doses over a period of time enhancing outcomes [8–12].

Mitochondrial bioenergetics of dental pulp stem cells (DPSCs) is an emerging area of research in regenerative medicine. The mitochondrial function of DPSCs can enhance tissue regeneration [13]. DPSCs have been reported to have a higher mitochondrial respiratory capacity than mesenchymal stem cells derived from bone marrow and adipose tissue [14]. An increase in oxygen respiratory rate of mitochondria of human DPSCs is consider important in their regenerative potential [15, 16]. Osteoblasts are specialized bone-formation cells important for healing following congenital diseases or surgery-induced bone loss [17]. Human Osteosarcoma MG-63 (MG-63) cells are popularly used for bone-related dental research [18]. The combination of osteoblastic properties, stability, proliferation rate, compatibility with experimental techniques, widespread use, and relevance to bone biology makes the MG-63 cell line widely used in cell culture models of bone research [19]. The cellular respiratory rate of MG-63 osteoblasts is modulated by the functionality of their mitochondria. This implies that alterations in mitochondrial function could potentially impact the cellular respiration and metabolic activity of these bone-forming cells [20].

Previous research has assessed the effect of PBM on mitochondria in 2D cultured cells [21]. 3D cell culture presents itself as a promising field for studying the effects of PBM on cells as it offers numerous advantages by closely mimicking in vivo physiological conditions, offering a superior emulation of the structure and function observed in human tissues and organs [22] and accurately representing cell-cell and cell-matrix interactions [23]. 3D cultures, however, are complex and need standardization to ensure optimal results [24]. For example, the standard method for 3D cell culture is hydrogel, which is a distinctive category of biocompatible scaffold resembling the extracellular matrix [25]. A limitation of this technology is that the thickness of these hydrogels could impact the effect of cellular modulators that could affect mitochondria inside the cells. A study conducted by Bikmulina et al. (2020) reported that near-infrared PBM (840-nm) in hydrogel-based structures that were 3 mm thick boosted cellular metabolism and promoted cell growth [24]. Curvello et al. (2021) demonstrated that hydrogels allowed for the recovery of cell clusters after passage, extraction of intact RNA, and preservation of significant viability for up to 4 days [26]. Hydrogels enable complete encapsulation of cells and growth factors while promoting cell proliferation and differentiation [27]. In a recent systematic review of the literature, we noted the positive effects of PBM on osteoblast-like cells, enhancing their proliferation and underscoring intracellular activities [28]. However, a significant lack of consensus was observed due to incomplete or inaccurately reported data [29]. Here, we describe a novel study with the aim to identify, the influence of 660 and 808-nm PBM treatments on mitochondrial oxygen consumption of MG-63 osteoblast cells in 3D cultures.

## Materials and methods

This study was approved by the Griffith University (Australia) human research ethics committee under reference number GU 2020/582 and all patients signed an informed consent form.

### 3D culture

MG-63 osteoblast cell line (MG-63) was cultured in the Dulbecco’s Modified Eagle/Ham’s F12 medium (DMEM/F12) (GIBCO; Invitrogen, Carlsbad, CA, USA) containing 10% fetal calf serum and 1% penicillin-streptomycin solution in standard conditions of 37 °C, 5% CO2. At confluence, cells were trypsinized with 0.05% trypsin/EDTA solution and passaged after 4 days.

### Preparation of spheroids

**Fig. 1:**
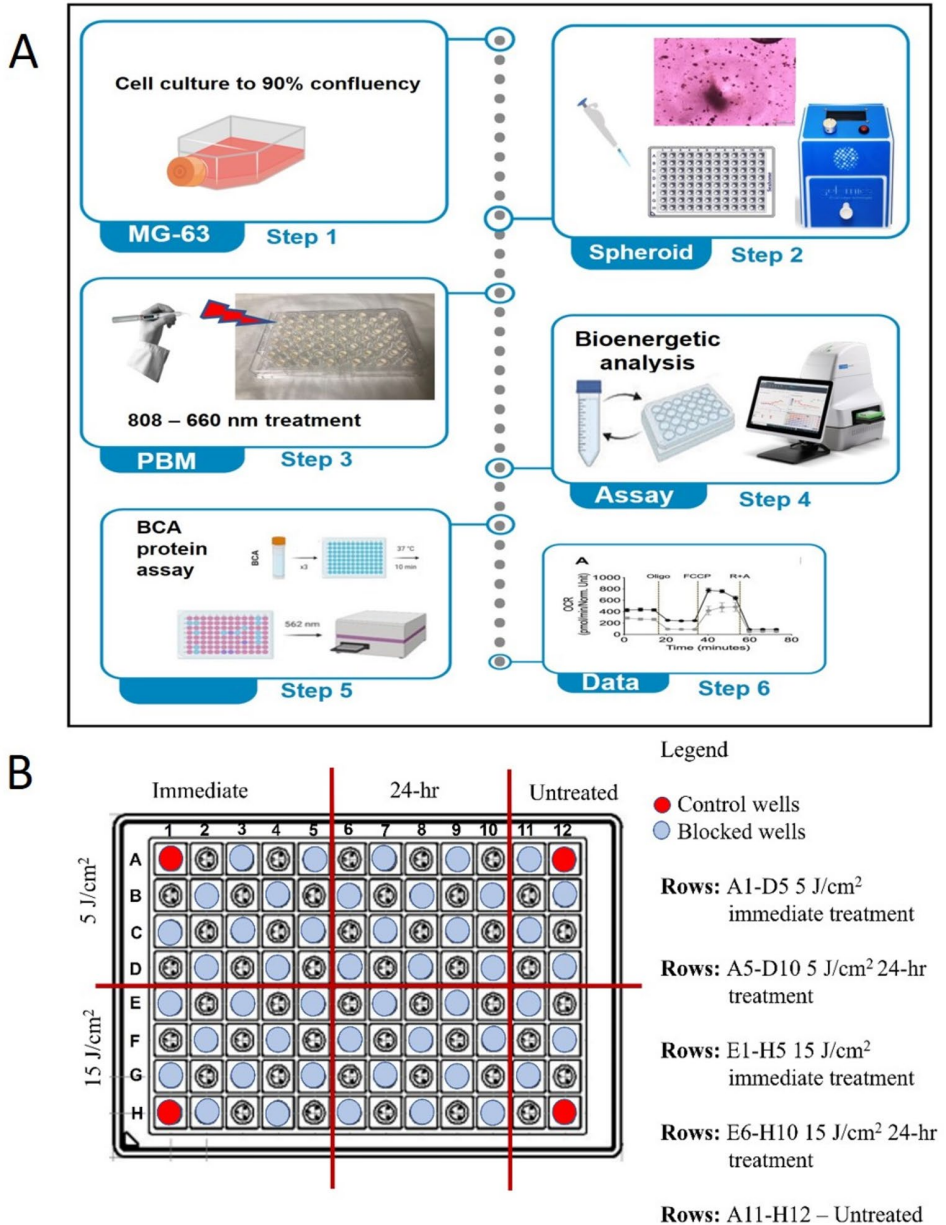
**(A)** Schematic flowchart for the isolated MG-63 and ATP assay using the Seahorse XFe96 Analyser. The real-time analyzer measures metabolic responses of MG-63 to modulators of Mito Stress Assay (MST). Basal OCR and extracellular acidification rates / proton production rates (PPR) were calculated using 2-minute mix and 4-minute measure cycles. Cycles of Oligomycin A with cycles of 4 as penetration within the spheroid to allow an observed effect. **(B)** Agilent Seahorse XFe96 Spheroid Microplate design showing space for cut steel straw to block wells

3D cell culture was generated using fish gelatin called Lunagel™ Photocrosslinkable Extracellular Matrix (Gelomics, QLD, Aust.). For the profiling of mitochondrial stress test following the PBM treatment, MG-63 osteoblast cells were first optimized for matrix low stiffness at 3.5 kPa by placing the cell plate in the Photocrosslinker (LunaCrosslinker™, Gelomics, Brisbane, Australia) for 2 min. To measure oxygen consumption rate (OCR) and extracellular acidification respiration (ECAR), the development of spheroids requires 7 days to form and is visibly evident in the wells before and after the assay shown in Fig. 1A. Spheroid movement is shown by a sudden decrease in OCR and ECAR results. Spheroids need to be stable as they are constantly injected with compounds, and oligomycin requires more time to penetrate the entirety of the spheroid.

### Optimisation of spheroidal microplate and PBM treatment

The 96-well microplate was divided into six treatment groups with four Control shown in Fig. 1B. To ensure there was no transmission of laser energy between wells, precision-cut steel straw was used between adjacent wells (Fig. 1B). The microplate wells were coated with Geltrex™ LDEV-Free hESC-qualified reduced growth factor basement membrane matrix (Gibco™, USA, #A1413201) following the manufacturer’s guideline. This forms a supportive gel layer in each well. Cells encapsulated in the hydrogel were then seeded onto the coated wells, and the microplate was then placed in the LunacrossLinker™ to set the LunaGel™. This step creates a stable environment, supporting cell attachment and growth.

A 7 mm diameter Optical Cap-B laser probe (Konftec™, Klas-DX, Taiwan) was used to deliver laser 660-nm (KLAS-DX61) and 808-nm (KLAS-DX84) energy directly over each well with no space between the probe and the well orifice. The laser was set at 5 or 15 J/cm^2^ (corresponding to 33 or 100_ S_) on the laser control handpiece. A power meter (Starbright by OPHIR^®^, Israel) with a photodiode sensor (PD300-1 W, Ophir^®^, Israel) was used to assess power output (Table 1). Controls (no treatment/placebo) for both 5 and 15 J/cm^2^ groups were undertaken by placing the uninitiated laser probe over the wells for 33s and 100s each treatment group. All treatment groups and control groups were then assessed immediately (15 min) or 24 h following PBM irradiation.

**Table 1:** Specific diode laser settings applied in the study

Parameters	Value	Value
Type of laser	808 nm Diode	660 nm Diode
Laser Manufacturer	Konftec™ Klas-DX KLAS-DX61	Konftec™ Klas-DX KLAS-DX84
Mode	Continuous	Continuous
Method of application	Non-contact	Non-contact
Delivery system	Optical multicore glass rod	Optical multicore glass rod
Power	230 mW	110 mW
Panel Setting	5–15 J/cm^2^	5–15 J/cm^2^
Power meter reading	6.6–22 J	3.7–11.35 J
Photon Fluence	9.9/33 p.j/cm2	7.03/21.56 p.j/cm2
Einstein Dose	2.47/7.3	1.34/4.65
Spot size mm	Optical Cap-B laser probe 7 mm	Optical Cap-B laser probe 7 mm
Spot area cm^2^	0.38 cm^2^	0.38 cm^2^
Duration	33 & 100 s	33 & 100 s
Pulse Frequency	3.70 Hz	4.94 Hz

### Cellular bioenergetics (Seahorse XFe96)

Mitochondrial respiration was assessed using the Seahorse XF96e Analyzer (Fig. 1). MG-63 cells were plated in Seahorse XFe 96 microplates (Agilent Technologies, Santa Clara, CA, USA) at a density of 1.5 × 10⁶ cells/mL, with 15,000–22,500 cells per well. After seven days, cells formed spheroids in LunaGel™. The XFe96 Sensor Cartridge was hydrated overnight with ultrapure water and incubated for 45–60 min with XF Calibrant solution at 37 °C in a CO2-free incubator. Cells were washed and incubated with XF Assay medium (pH 7.4). Seahorse assay medium (37 °C) contained 10 mM D-glucose, 1 mM sodium pyruvate, and 200 mM glutamine (Agilent Technologies #103579-100). Mito Stress (Agilent #103325-100) stock agents were injected at 10× concentrations according to manufacturer guidelines. OCR data were normalized to protein concentration and used to calculate mitochondrial metrics using validated techniques [30].

### Cell viability using almar blue assay

The hydrogel mix of cells was seeded into a 96-well plate containing 100 µL/well of cell culture medium. The plate was placed in an incubator overnight in a 37 °C incubator. The laser 808-nm (KLAS-DX84) and 660-nm (KLAS-DX61) energy at 5 J/cm^2^ (33s) was directly used over each well and control received no laser treatment. The cell viability reagent (AlamarBlue™ HS Cell Viability Reagent, cat. #A50100 Invitrogen, CA, USA) was warmed to room temperature, and 1/10th volume of cell viability reagent was added directly to cells in culture medium according to manufacturers’ guidelines and incubation for 4 h at 37 °C in a cell culture incubator. After an incubation period, fluorescence emitted by the AB was measured using a Tecan Genios microplate reader at the specified excitation and emission wavelengths to monitor the absorbance of reagent at 570-nm, using 600-nm as a reference wavelength for normalization.

### Cell recovery

Cell retrieval solution was prepared by mixing one part of a 10x concentrated stock solution of LunaGel™ - (Cell Recovery Kit- #SKU0015, Gelomics, Brisbane, Australia) with nine parts of a provided specialized cell retrieval buffer. Culture medium was removed from the spheroid-containing wells, and a 0.25µL of the diluted cell retrieval solution was added to each well of a 96-well plate. The plate was then incubated at a temperature of 37 °C for a period ranging between 30 and 40 min, or until complete digestion is observed. After incubation, two volumes of culture medium are added to re-suspend the cells. The samples were then centrifugated 500xg for 3 min as per standard procedures and supernatant discarded.

### BCA protein normalisation

Protein concentrations were assessed utilising the BCA (Pierce BCA Protein Assay Kit, Thermo Scientific cat.#23227, USA) assay following specific instructions and safety precautions as outlined in the manual were strictly followed. To improve the accuracy of the results, control samples were run, and replicates were performed.

### Statistical analysis

All data were analyzed using GraphPad Prism version 8.4.1 (San Diego, CA, USA). Statistical analyses were performed to evaluate differences in mitochondrial metabolism among treatment groups. Data were normalized to account for variations in sample size, and percent changes relative to the control were calculated to assess changes in basal and maximal respiration rates, providing insights into dose-response effects. A statistician assessed the data for normality using Kolmogrov-Smirnov and Shapiro-Wilk test. For normally distributed data, t-tests were used, while for non-normally distributed data, the Mann-Whitney U test was applied. To compare multiple groups, one-way ANOVA with post-hoc Kruskal-Wallis test was used, where appropriate. Statistical significance was determined using a p-value (*p* ≤ 0.05).

## Results

To ensure dose standardization between wells the transmission of laser energy between wells through the bottom of the plate was assessed in addition to precautions taken to prevent transmission between the wall of the well. These results of this assessment indicated that both the laser transmitted through the media and gel was extremely low for 660 nm (43.6 mW) and 808 nm (70.8 mW) lasers used in the experiment, with no energy being recorded in any adjacent well.

This 3D cell culture study used Lunagel™ Photocrosslinkable Extracellular Matrix (Gelomics, Brisbane, Australia) with a stiffness of 3.5 kPa, selected to mimic the in vivo microenvironment. This stiffness supported relevant cell behaviours, including adhesion, proliferation, and differentiation, while enabling effective delivery of mitochondrial stress test modulators like Oligomycin for optimal experimental conditions.

**Fig. 2:**
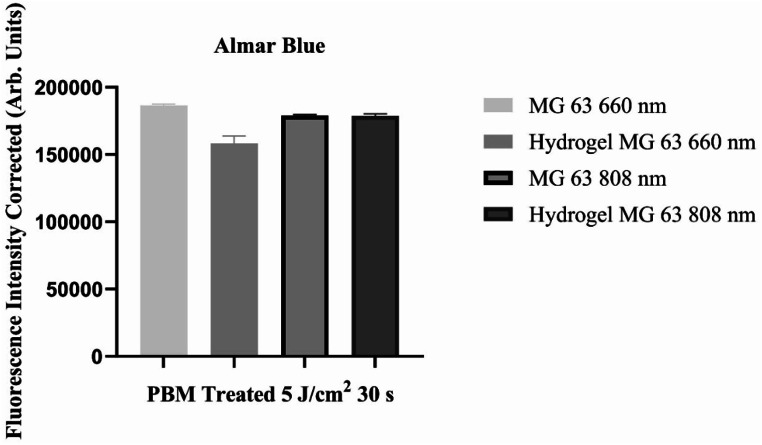
Shows the results of viability testing showing viability of cell in hydrogel and with laser

Alamar blue (AB) assay for cell viability was obtained after compensation of the AB solution concentration for spheroids grown with no gel and in fish gelatine (LunaGel™). The Alamar blue assay showed that cell in treated with 660-nm and 808-nm laser both with and without hydrogels remained viable shown in Fig. 2.

**Fig. 3:**
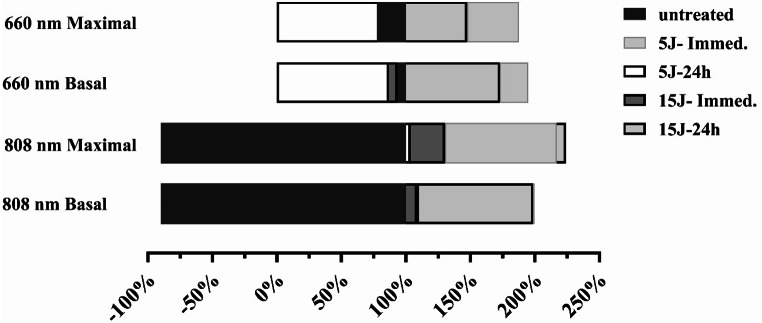
**(A)** Basal Respiration and **(B)** Maximal Respiration showing oxygen consumption rate (OCR) kinetics increased post-photobiomodulation, indicating trend for improved metabolism after 808-nm wavelength immediate and 24-hour treated cells at 5 J/cm^2^ and 15 J/cm^2^. The 660-nm PBM group showed a decrease in the immediate and 24-hour treated cells at 5 J/cm^2^ and 15 J/cm^2^. (sample size *n* = 9–10 and statistical significance for each result)

PBM treated spheroids with 660-nm and 808-nm showed a dose response in mitochondrial respiration for all laser irradiated treatment groups. Compared to untreated group the results showed that a single dose treatment does not statically affect the respiration rate for all treatment groups. However, trends indicate that the 808-nm laser treatment groups (Fig. 3a & b) showed an increased basal and maximal oxygen consumption rate compared with the effect being more pronounced for maximal respiration.

The 660-nm groups on the contrary showed a decrease in both basal and maximal respiration across all energy settings. The percentage change versus untreated control for basal respiration for 808-nm laser showed a notable decrease by -20% for 5 J/cm^2^ dose at 24-hrs and a substantial decrease of -30% for 15 J/cm^2^ dose at 24-hrs. The maximal respiration showed significant increase by 30% at 15 J/cm^2^ at 24-hrs and a smaller change at 16% for 5 J/cm^2^ dose at 24-hrs. The 660-nm laser treatment for basal respiration decreased slightly with the highest reduction by -13% at 5 J/cm^2^ at 24-hrs compared to 808-nm treatment. The maximal respiration changes were minor with no significant increased change indicating less effective impact on mitochondrial capacity compared to 808-nm laser. Raw data provided in Supplementary Table 1.

## Discussion

This study investigated the effects of PBM on MG-63 osteoblast cells using innovative 3D cell culture techniques. The Seahorse Extracellular Flux Analyser (XFe96) performs real-time monitoring of oxidative phosphorylation (OXPHOS) in living cells [31]. This study identified changes in oxygen consumption rate following PBM therapy, revealing insights into mitochondrial activity. It is important to note that several factors could influence the absorbed PBM dose by cells [32]. Variations in absorbed dose are reported to affect clinical outcome and hence, the need to optimize dose [28]. Wavelengths ranging from 600 to 950-nm are commonly preferred for their greater ability to penetrate tissue optically [1, 28, 32].

This study considered several optimization measures, including a hydrogel stiffness of 3.5 kPa and a cell density of 1.5 × 10⁶ cells/mL. Hydrogel optimization is crucial, as uneven thickness can cause scattering and variations in light absorption, reducing exposure to cells at deeper depths [5]. In the pilot phase, oligomycin diffusion was slightly delayed, likely due to the hydrogel’s stiffness and its smaller pore size. To address this, measurement time on the Seahorse analyzer was extended. Based on prior experience, a cell density of 1.5-2 × 10⁶ cells/mL is optimal for encapsulating MG-63 cells in LunaGel™, which uses Photocrosslinking™ to ensure consistent matrix stiffness across experiments.

The Mito Stress Test reveals key aspects of cellular respiration, including ATP production, mitochondrial membrane leaks, spare capacity, and non-mitochondrial oxygen consumption. Enhanced OCR kinetics suggest improved mitochondrial bioenergetics [33]. We observed that 808-nm light promoted greater mitochondrial OCR compared to 660-nm light. Eroglu et al. (2021) demonstrated that 808-nm light at 3 J/cm² could rejuvenate aged mesenchymal stem cells by targeting mitochondrial chromophores and penetrating deep tissues [34]. Consistent with this, our study found that PBM with an 808-nm laser increased the maximal respiratory rate), with immediate treatments producing a more pronounced spike in OCR. This indicates that there is an optimal dose range that maximizes the metabolic regulatory effects of PBM on osteoblasts and there is evidence that each lineage likely has an optimal PBM dose [35]. Gene expression could capture the steady state over longer time frames, but mechanistic PBM photobiological responses are very dynamic and will likely require other approaches.

A study by Diniz et al. (2018) showed cell proliferation and stem cell differentiation using 660-nm PBM in the 3D model using DPSCs encapsulated in the hydrogel, with energy densities of 3 J/cm^2^ (4 s) and 5 J/cm^2^ (7 s) [36]. Comparable outcomes were observed with a wavelength of 630-nm PBM when utilizing lower energy densities, specifically at 1.5–2.5 J/cm^2^, on bone marrow mesenchymal stem cells [37]. The authors propose that increases in mitochondrial activity occurred under single-dose light stimulation, which induces cell proliferation [37]. In a 3D cell culture, it was reported that cells containing higher mitochondrial content exhibit a greater real-time mitochondrial response [38]. The current study using 3D cell culture to assess mitochondrial activity noticed no increase in mitochondria’s basal or maximal oxygen consumption when using 660-nm PBM. Asnaashari et al. (2022) demonstrated that 660-nm and 808-nm diode lasers, operating at an energy density of 4.1 J/cm^2^, elevated the OCR of mitochondria in a 2D model employing hDPSCs, suggesting heightened mitochondrial functionality associated with improved cell viability and proliferation [39]. However, these findings contradict our results obtained with the 660-nm laser at 3.7/11.35 J/cm^2^, indicating a reduced mitochondrial activity and the need to explore alternative parameters for the 660-nm laser in the first 24 h.

In general, influences of red or Near Infrared (NIR) as low as 3–5 J/cm^2^ have shown to be beneficial in vivo, but a large dose of 50–100 J/cm^2^ will lose the beneficial effect and may even become detrimental [39]. One study by Hsieh et al. (2023) investigated 808-nm wavelength daily 15-minute increments and showed bolstered resistance to mitochondrial inhibitors and uncoupling agents through two separate mechanisms [40]. The light at 1.40 mW/cm^2^ accelerated cell growth and increased the percentage of the cell cycle. Meanwhile, light at 1.95 mW/cm^2^ amplified oxidative phosphorylation at the levels of RNA, protein, and functionality while also increasing the percentage of mitochondria with a network-like structure [41].

It is important to note that despite setting the laser fluency at 5 and 15 Joules, the power meter readings for the two wavelengths differed (Table 1). The 808-nm wavelength recorded higher fluency values of 6.6 J and 22 J, whereas the 660-nm wavelength recorded lower fluency values of 3.7 J and 11.35 J, respectively, compared to the settings on the laser device. In general, the fluency for the 808-nm laser was approximately twice as large as that for the 660-nm laser under each set parameter. However, since the photon energy for the 660-nm laser (1.9 eV) is higher than that of the 808-nm laser (1.5 eV), the photonic fluency for the 660-nm laser was only about a quarter lower (Table 1). Besides the inherent differences in photon energy of individual wavelength that affect biological penetration depths, there are differences in their quantum efficiency for energy transfer [42, 43]. Moreover, selective biological chromophores could also contribute to these differences that are being actively investigated [44] Despite these complexities, the study found that the 660-nm laser was associated with a decrease in maximal respiration rate. This suggests that either the fluency used was not optimal for the observed effect or that other laser parameters need further exploration.

To optimize therapeutic effects, careful consideration of various experimental parameters is essential. These include the characteristics of the irradiated biological material, penetration depth, exposure duration, etc(ref). Additionally, the type of light source (LEDs vs. lasers) and the wavelength (ranging from 808 to 980 nm) are crucial factors that influence the overall outcome [10, 43, 45, 46]. Achieving optimal photobiomodulation (PBM) dosing is particularly important and should be tailored based on clinical presentations. This study addresses a gap in the current literature regarding the effects of PBM on mitochondrial bioenergetics, particularly in the context of 3D cell culture systems. We anticipate that our approach will enhance both experimental outcomes and assay reliability, providing a more nuanced understanding of metabolic compartmentalization and oxygen consumption in vitro.

## Conclusion

This study used MG-63 in 3D culture to provide an insight into mitochondrial bioenergetics post-PBM. The enhancement of the maximal respiratory rate observed with 808-nm PBM could promote greater mitochondrial activity and hence enhance energy need to supporting bone cell proliferation, differentiation, and overall bone healing. The decrease in oxygen consumption rate (OCR) induced by 660-nm PBM may indicate a more inhibitory or regulatory effect on mitochondrial function. This could be leveraged for applications were reducing excessive metabolic activity or modulating cell function is important such as in controlling pathological osteoblast activity. Future studies should explore different doses and durations to optimize the effect of PBM in 3D cultures.

## Electronic supplementary material

Below is the link to the electronic supplementary material.

Supplementary Material 1

## Data Availability

No datasets were generated or analysed during the current study.
