# Supplementary material for: Assessment of the influence of 660 and 808-nm PBM treatments on mitochondrial oxygen consumption of MG-63 osteoblast: a 3D cell culture study
Source: Lasers Med Sci. 2025 Feb 12;40(1):84. doi: 10.1007/s10103-025-04349-3 (PMC11813955; doi:10.1007/s10103-025-04349-3)
Supplement: Supplementary file 1 — Supplementary Material 1 [file 10103_2025_4349_MOESM1_ESM.pdf]

Supplementary Table 1

Table 1. PBM 810 nm and 660 nm basal and maximal respiratory rates.

Laser			untreated	5J Immed.	5J 24-hrs	15J Immed.	15J 24-hrs
**808 nm**	**Basal Respiratory rate**	**Mean**	100.0	99.5	86.9	108.7	89.9
	**Maximal Respiratory rate**	**Mean**	100.0	116.8	103.7	130.3	93.8
**660 nm**	**Basal Respiratory rate**	**Mean**	100.0	94.8	86.8	93.4	79.5
	**Maximal Respiratory rate**	**Mean**	100.0	87.7	82.0	77.6	69.9
